# Impacts of Self-Esteem and Self-Perceived Burden on Health-Related Quality of Life Among Patients with Ovarian Cancer: Does Age Matter?

**DOI:** 10.3390/curroncol33010023

**Published:** 2026-01-01

**Authors:** Lei Dou, Li Liu, Zhichen Liu, Yajing Wang, Hui Guo, Yiqun Xiao, Meizhu Pan, Yuli Song, Hui Wu, Yi Zhang

**Affiliations:** 1 Department of Gynecology, The First Affiliated Hospital of China Medical University, Shenyang 110001, China; 2016110181@cmu.edu.cn (L.D.); lliu09@cmu.edu.cn (L.L.); wyj1625694738@163.com (Y.W.); 2025110229@cmu.edu.cn (M.P.); song_yuli@163.com (Y.S.); 2 Department of Social Medicine, School of Health Management, China Medical University, Shenyang 110122, China; liuzhichen5329@163.com (Z.L.); guohchn@163.com (H.G.); psy11xyq@163.com (Y.X.)

**Keywords:** health-related quality of life, self-esteem, self-perceived burden, age difference, moderated mediation, ovarian cancer

## Abstract

**Background:** Ovarian cancer, the most lethal gynecologic malignancy, is characterized by a poor health-related quality of life (HRQoL). The present study examined the mediating role of self-perceived burden (SPB) in the impact of self-esteem on HRQoL and whether age moderated the associations among ovarian cancer patients. **Methods:** 203 patients effectively completed the Functional Assessment of Cancer Therapy-General (FACT-G), Rosenberg Self-Esteem Scale, and SPB scale, respectively. For the FACT-G, physical (PWB), social/family (SFWB), emotional (EWB), and functional well-being (FWB) were scored separately. **Results:** Significant mediation of SPB in the impacts of self-esteem on PWB (*a* × *b* = 0.074, 95% CI: 0.018, 0.153), EWB (*a* × *b* = 0.048, 95% CI: 0.001, 0.125), and FWB (*a* × *b* = 0.056, 95% CI: 0.009, 0.114) were revealed. Age positively moderated the impact of self-esteem on SPB (*β* = 0.159, *p* < 0.05), and the associations of SPB with PWB (*β* = 0.173, *p* < 0.05) and EWB (*β* = 0.240, *p* < 0.01), indicating a moderated mediation. **Conclusions:** Ovarian cancer patients’ self-esteem could improve the PWB, EWB, and FWB domains of HRQoL by reducing SPB. Age could attenuate SPB’s mediation in the impacts of self-esteem on PWB and EWB, indicating stronger impacts in younger patients. Clinical programs integrating components that strengthen self-esteem and reduce SPB may be particularly beneficial for younger women with ovarian cancer.

## Simple Summary

Ovarian cancer is the third most common gynecologic cancer and the second leading cause of death from gynecologic malignancy worldwide. Better health-related quality of life is an important predictor of improved prognosis. This study first demonstrated a moderated mediation model of quality of life involving self-esteem, self-perceived burden and patient’s age. Self-esteem could play positive roles on physical well-being, emotional well-being, and functional well-being via reducing self-perceived burden. The association between self-esteem and self-perceived burden, and the associations of self-perceived burden with physical well-being and emotional well-being, were gradually decreased with the increase in the patient’s age. Clinical programs integrating components that strengthen self-esteem and reduce self-perceived burden may be particularly beneficial for younger women with ovarian cancer.

## 1. Introduction

Ovarian cancer is the third most prevalent gynecologic malignancy and the second greatest cause of mortality from gynecologic cancer worldwide [1]. As the most lethal gynecologic cancer, ovarian cancer is often diagnosed at advanced stages [2,3]. Patients frequently have extensive sequelae of ovarian cancer and its treatment, such as physical and psychological symptoms, and impaired social function, which can seriously damage their health-related quality of life (HRQoL) [4,5].

Researchers have gradually focused on positive psychological resources for dealing with the suffering caused by cancer [6]. Self-esteem refers to people’s overall attitude toward the self: how to judge and evaluate themselves and give themselves value, worth, and respect as human beings [7,8]. As a positive psychological construct, self-esteem is vital in psychological adaptation, re-establishing social roles and functions among cancer patients [9]. Greater self-esteem can help patients cope with cancer or treatment-related stress by facilitating positive psychosocial responses, social support utilization, successful personal control, and well-being [10]. Among women with breast and cervical cancer, self-esteem was positively related to HRQoL [11,12]. Self-esteem partially mediated the association between perceived unsupportive behaviors of family/friends and psychological suffering among ovarian cancer patients [13]. The perception of femininity loss is a unique feature of gynecologic cancers compared with other cancer types in women. For many women, self-esteem is mainly based on the perception of their femininity [14]. However, evidence for the effect of self-esteem on HRQoL and its underlying mechanism in patients with ovarian cancer is lacking.

Self-perceived burden (SPB) is a term used to describe the care recipient’s sympathetic worry about how their care requirements will affect the caregiver, which may result in guilt and grief in the receiver [15]. Self-esteem is often considered an internal psychological resource that helps cancer patients reduce their SPB [16]. Despite the lack of direct evidence, several self-esteem-related concepts, such as self-stigma and self-efficacy, have been shown to relate to the level of sense of burden. A systematic review indicated that cancer stigma was negatively associated with self-esteem and self-efficacy and positively related to SPB [17]. According to earlier research, cancer patients frequently suffer from SPB, significantly lowering their HRQoL [18,19]. Among ovarian cancer patients, SPB could exacerbate psychological suffering by escalating loneliness [20]. Given the Chinese traditional culture, SPB may have an exceptionally critical impact on the HRQoL of ovarian cancer patients due to gender role norms. Chinese women are expected to put the family first and are self-sacrificing as the caregivers of their families [21]. Family members are the primary caregivers for ovarian cancer patients in conventional patient-caregiver interactions. Due to the reorganization of family responsibilities, women with cancer frequently feel overwhelmed by their caregivers or relatives [22]. Nevertheless, the impact of SPB on HRQoL has not been determined in ovarian cancer patients. In addition, SPB mediated the negative impact of self-stigma on HRQoL among Chinese American breast cancer survivors [19]. SPB also acted as a mediator in the associations of psychological resilience and stigma with HRQoL in cancer patients [23]. Consequently, it is reasonable to assume that SPB is a mediator in investigating the mechanism connecting self-esteem and HRQoL among ovarian cancer patients.

Age could act as a potential moderator on the underlying links between self-esteem, SPB, and HRQoL in ovarian cancer patients. Because of the differences in social roles and functions, women patients have age-specific self-evaluation and a sense of burden corresponding to cancer and treatment outcomes [24]. Once cancer occurs in the early life cycle stage, younger patients must face more pressures from marriage and childbirth, family roles, and future expectations. Young women with gynecologic cancer will face additional stress, mainly from fertility [25] and/or sexual function [26]. Ovarian cancer is generally considered a disease that primarily compromises older women’s health and is combined with various chronic diseases [27]. Emotional sensitivity to cancer-related distress exhibits a striking contrast between younger and older women, modulated by age-specific psychosocial and developmental priorities [28,29]. Therefore, the cognitive and emotional problems induced by ovarian cancer and its treatment may have a more profound negative impact on the HRQoL of younger patients. Age has been found to moderate the relationship of cognitive function with post-traumatic stress in women with metastatic breast cancer, and younger patients are susceptible to stress [30].

**Figure 1:**
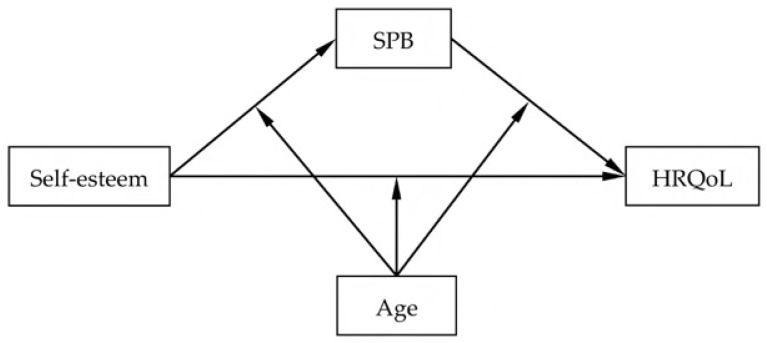
Hypothetical model. SPB: self-perceived burden; HRQoL: health-related quality of life.

Currently, no previous study has simultaneously examined the mediation of SPB and the moderation of age in the relationship between self-esteem and HRQoL among women with ovarian cancer. To address this scientific gap, the present study examined a moderated mediation model, as summarized in Figure 1. We hypothesize that: (1) self-esteem is positively associated with HRQoL, (2) self-esteem is negatively associated with SPB, (3) SPB has a negative association with HRQoL, (4) SPB acts as a mediator between self-esteem and HRQoL, and (5) age moderates the associations within this mediational pathway, including self-esteem–HRQoL, self-esteem–SPB, and SPB–HRQoL.

## 2. Materials and Methods

### 2.1. Study Design and Sample

From March to June 2021, patients with primary ovarian cancer were enrolled in a single-center, cross-sectional survey at the Department of Gynecology of the First Affiliated Hospital of China Medical University. Those who met the following criteria could be enrolled in the study: (1) those who had to be at least 18 years old, (2) those who had an ovarian cancer pathology diagnosis, (3) those who received surgery, adjuvant treatment, or palliative care for at least 1 month prior to our survey, (4) those who were aware of their cancer diagnosis, and (5) those who had a normal cognitive function to fill out the questionnaire. If a participant had a history of psychiatric disease, cognitive impairment, or another kind of malignancy, she was disqualified from the study.

After discussing each patient’s eligibility, the attending physicians recruited all eligible patients through a face-to-face or telephone consultation. Inpatients and outpatients were given a face-to-face interview to complete a structured questionnaire, whereas community patients who had finished their hospital therapy were given a telephone interview. To ensure standardization between face-to-face and telephone interview modes, we implemented the following comprehensive strategies: (1) the identical questionnaire was used; (2) all interviewers received standardized training emphasizing neutral probing; (3) the questionnaire and interview protocol were piloted in both modes. Trained investigators were responsible for providing patients with non-suggestive assistance in reading the items and entering their responses. Clinical information was taken from the medical records of each participant. The study was conducted in accordance with the Declaration of Helsinki, and approved by the Ethics Committee of China Medical University. All procedures performed in this study complied with the applicable guidelines and regulations. Prior to data collection, informed consent was acquired from each participant through either written or verbal means.

A total of 302 eligible patients were approached for participation. Of these, 88 declined, citing severe physical symptoms, concerns about the burden of participation, or a perceived lack of necessity for psychological assessment. Finally, two hundred fourteen qualified patients were accepted to participate in the study. They completed the questionnaires, while eleven responses were excluded from the final analysis because of missing data (5 for HRQoL, 2 for self-esteem, 3 for SPB, and 1 for clinical information). Accordingly, the participating patients provided 203 (94.9%) effective responses.

### 2.2. Measures

#### 2.2.1. Outcome Variable (HRQoL)

HRQoL was measured using Version 4 of the Functional Assessment of Cancer Therapy-General (FACT-G), a specific measuring instrument for cancer patients with good validity worldwide [31]. The FACT-G includes 27 questions that are designed to assess four domains of cancer-targeted HRQoL: physical well-being (PWB, 7 items, e.g., I have a lack of energy, and I am forced to spend time in bed), social/family well-being (SFWB, 7 items, e.g., I get emotional support from my family, and My family has accepted my illness), emotional well-being (EWB, 6 items, e.g., I feel sad, and I worry about dying), and functional well-being (FWB, 7 items, e.g., My work [include work in home] is fulfilling, and I am enjoying my usual leisure pursuits). For scoring the FACT-G’s items, a 5-point Likert scale is used from 0 (not at all) to 4 (very much). The summed scores of the PWB, SFWB, and FWB subscales range from 0 to 28, while the summed score of the EWB subscale ranges from 0 to 24. Better HRQoL is indicated by a higher score. The current study’s PWB, SFWB, EWB, and FWB subscales had Cronbach’s alpha values of 0.87, 0.85, 0.81, and 0.90, respectively.

#### 2.2.2. Independent Variable (Self-Esteem)

The Rosenberg Self-Esteem Scale (RSES), a robust measuring instrument in Chinese cancer populations, was used to measure self-esteem [32]. The RSES contains ten items that are designed to assess self-worth or self-acceptance, with five positive (e.g., On the whole, I am satisfied with myself, and I feel that I have a number of good qualities) and five negative items (e.g., I feel I do not have much to be proud of, and I certainly feel useless at times). Responses to all items were recorded using a 4-point Likert scale, with anchors set at 1 for strongly disagree and 4 for strongly agree. The range of the summed RSES score is 10 to 40 after reverse-scoring the negatively worded items. The level of self-esteem increases with the RSES score. The RSES’s Cronbach’s alpha value in our sample was 0.85.

#### 2.2.3. Mediator (SPB)

Patient’s self-perception of the financial, emotional, and physical burden on their caregivers was assessed using the Self-Perceived Burden Scale (SPBS) [15,33]. The SPBS contains ten items, e.g., Worry caregiver’s health could suffer, and Feel guilty about the demands I make. A Likert scale of 1 to 5 is used to grade the SPBS items, with 1 denoting never and 5 denoting often. The SPBS’s total score might be between 10 and 50. A higher score denotes a greater SPB level. The SPBS has been widely used in China for various cancer patients [18,23]. The current sample’s Cronbach’s alpha for the SPBS was 0.95.

#### 2.2.4. Moderator (Age)

Age was collected as a continuous variable and treated as a continuous variable in moderated mediation analysis. Age was presented in four categories for descriptive purposes: ≤45, 46–55, 56–65, and >65 (years).

#### 2.2.5. Potential Confounders (Demographic and Clinical Characteristics)

For demographic characteristics, marital status was classified into two categories: married/cohabited and single/divorced/widowed/separated. Education was divided into five groups: primary school or below, junior high school, senior high school, junior college, and college or above. Place of residence was clustered into two categories: urban and rural. Annual household income was divided into four groups: <30,000, <80,000, <120,000, and ≥120,000 (Chinese Yuan, CNY).

For clinical characteristics, disease status was split into new-onset and relapse. Time since cancer suffering was divided into five groups: ≤1, ≤2, ≤3, ≤4, and >4 (years). Patients’ cancer stages were determined using the 2014 International Federation of Gynecology and Obstetrics (FIGO) staging criteria. Surgery style was classified into three categories: minimally invasive surgery, laparotomy, and no operation. Data concerning patient history of chemotherapy, chronic comorbid conditions, familial cancer, and pregnancy were collected, with each variable being consolidated into two categories for analysis.

### 2.3. Statistical Analysis

Independent sample *t*-test or one-way analysis of variance was used to identify group differences. Pearson correlation evaluated the linear relation between two continuous variables. Two sequential steps proceeded to test the hypothesized moderated mediation using the PROCESS macro v4.0, which is a widely adopted and robust tool for path analysis-based conditional process modeling. It is particularly appropriate as it allows for the simultaneous estimation of mediation and moderation within a single, integrated framework. First, we used Model 4 to establish the mediating role of SPB. Subsequently, to examine whether this mediation effect was moderated by age (treated as a continuous variable), we employed Model 59, which is specifically designed for testing a conditional process model with a moderator acting on all the mediational pathways. Self-esteem was modeled as the independent variable. The dependent variables were the four domains of HRQoL (PWB, SFWB, EWB, and FWB). Meanwhile, the mediator was SPB, and the moderator was age as a continuous variable. The *Z*-scores of the four variables were calculated to eliminate scale disparities in the regression analyses. Covariates (potential confounders) were selected based on a statistically significant relation with either the mediator (SPB) or the outcome variable (HRQoL) in preliminary univariable analyses. They were included as covariates in the PROCESS models to control for their potential influence. A mediation is statistically significant when the 95% confidence interval (95% CI) of the mediation does not contain the null hypothesis value, with a bootstrapping sample of 5000. Moreover, simple slope analysis addressed continuous age’s moderation using mean-centering variables at specific levels of age: low (−1 standard deviation [SD], mean, and high [+1 SD]). Questionnaires with any item-level missing data were excluded from the final analysis. SPSS version 21.0 (IBM, Asia Analytics Shanghai) was adopted for statistical analysis. A *p*-value, less than 0.05 for a two-tailed test is thought to be statistically significant.

## 3. Results

### 3.1. Demographic and Clinical Characteristics

Table 1 displays the demographic and clinical characteristics of our sample. Patients’ ages ranged from 18 to 76 years, with a mean of 54.4 and a SD of 10.2. One hundred and eighty-two (89.7%) subjects were married or cohabited, and 130 (64.0%) subjects were urban residents. Regarding clinical characteristics, 179 (88.2%) patients were diagnosed with new-onset ovarian cancer. One hundred and forty-five (71.4%) subjects had a time since cancer suffering of ≤3 years, and 132 (65.0%) patients reported at least one chronic comorbidity.

### 3.2. Descriptive Statistics and Correlations

Table 2 displays descriptive statistics for the means, SDs, and correlations of self-esteem, SPB, and HRQoL domains. Self-esteem demonstrated a positive correlation with PWB, SFWB, EWB, and FWB. Conversely, it exhibited an inverse correlation with SPB (*r* = −0.353, *p* < 0.01). Furthermore, SPB was negatively correlated with PWB, EWB, and FWB.

### 3.3. Mediating Role of SPB

Table 3 shows association coefficients including *c* (the total impact of self-esteem on HRQoL), *a* (the impact of self-esteem on SPB), *b* (the impact of SPB on HRQoL), *c′* (the direct impact of self-esteem on HRQoL with SPB as a mediator), and *a* × *b* (the mediation of SPB). In the univariate analyses of screening covariates, marital status, residence, disease status, time since cancer diagnosis, and comorbidity were significantly related to SPB or the four domains of HRQoL (*p* < 0.05). The results are displayed in further detail in Supplementary Table S1. These variables were incorporated as covariates in the regression models to control for their effects (using the Model 4 of the PROCESS macro). Significant mediating roles of SPB in the impacts of self-esteem on PWB (*a* × *b* = 0.074, 95% CI: 0.018, 0.153), EWB (*a* × *b* = 0.048, 95% CI: 0.001, 0.125), and FWB (*a* × *b* = 0.056, 95% CI: 0.009, 0.114) were revealed. SPB’s mediation accounted for 21.08%, 13.79%, and 12.99% of the total impact of self-esteem on PWB, EWB, and FWB, respectively.

### 3.4. Moderated Mediation

**Figure 2:**
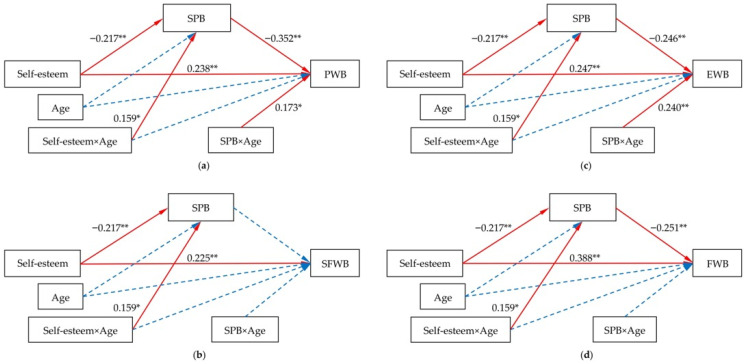
Moderated mediation model for the associations among self-esteem, SPB, age and HRQoL. (**a**) Moderated mediation model for PWB; (**b**) Moderated mediation model for SFWB; (**c**) Moderated mediation model for EWB; (**d**) Moderated mediation model for FWB. Standardized path coefficients were displayed. Marital status, place of residence, disease status, duration of suffering from cancer and chronic comorbidity were adjusted. Statistically significant paths were indicated with solid red lines and non-statistically significant paths with dashed blue lines. *, *p* < 0.05 (two-tailed), **, *p* < 0.01 (two-tailed). SPB: self-perceived burden; PWB: physical well-being; SFWB: social/family well-being: EWB: emotional well-being; FWB: functional well-being; HRQoL: health-related quality of life.

Figure 2 presents the tested moderated mediation models (using Model 59 of the PROCESS macro). The models explained substantial variance, particularly in PWB (41.1%) and FWB (31.3%). No significant multicollinearity was detected, with all variance inflation factor (VIF) values well below the threshold of 5 (maximum = 1.420). Then, statistical powers were calculated with a post hoc approach using *R*^2^, sample size (203), alpha (0.05), and predictor’s number (10), and they exceeded 0.955 for all models.

#### 3.4.1. Moderating Roles of Age on Mediational Paths

Figure 2 presents age as a significant moderator. Firstly, age positively moderated the impact of self-esteem on SPB (*β* = 0.159, *p* < 0.05). The interaction of self-esteem and age accounted for an additional 2.24% of the variance of SPB. Secondly, age also showed a significant moderation on the impacts of SPB on PWB (*β* = 0.173, *p* < 0.05) and EWB (*β* = 0.240, *p* < 0.01), respectively. The interaction of SPB and age accounted for an additional 1.84% and 3.53% of the variance of PWB and EWB, respectively. This indicates that the strengths of these key impacts were not fixed but varied with the patient’s age.

**Figure 3:**
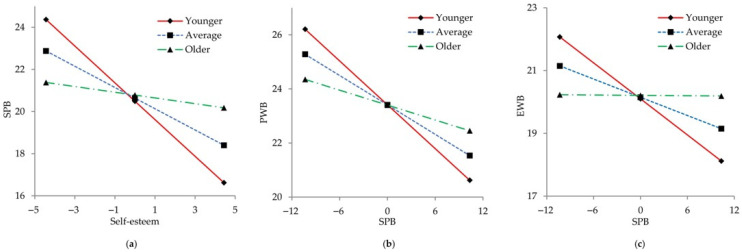
Moderating roles of age. (**a**) Moderating the impact of self-esteem on self-perceived burden; (**b**) Moderating the impact of self-perceived burden on PWB; (**c**) Moderating the impact of self-perceived burden on EWB. Younger group, −1 SD; Average group, the mean; Older group, +1 SD. Marital status, place of residence, disease status, duration of suffering from cancer and chronic comorbidity were adjusted. It indicates the younger group in red, the average group in blue, and the older group in green. SPB: self-perceived burden; PWB: physical well-being; EWB: emotional well-being; SD: standard deviation.

Figure 3 plots the moderation of age to interpret how the impacts change across age groups intuitively. The conditional effects are presented at low (−1 SD), mean, and high (+1 SD) levels of age. The impact of self-esteem on SPB gradually decreased with age (younger group: *β* = −0.375, *p* < 0.01; average group: *β* = −0.217, *p* < 0.01; older group: *β* = −0.058, *p* = 0.545). Correspondingly, the impact of SPB on PWB and EWB also showed a decreasing pattern with age (PWB: younger *β* = −0.525, *p* < 0.01; average *β* = −0.352, *p* < 0.01; older *β* = −0.179, *p* = 0.046. EWB: younger *β* = −0.485, *p* < 0.01; average *β* = −0.246, *p* < 0.01; older *β* = −0.006, *p* = 0.956).

#### 3.4.2. Moderating Roles of Age on Mediations

As shown in Table 4, the mediating roles of SPB in the impacts of self-esteem on PWB and EWB gradually decreased with age. Together, these patterns indicate that the entire psychological pathway—from self-esteem, through reduced SPB, to improved PWB and EWB—was most robust among younger patients. For older patients, this specific chain of influence was markedly weaker or non-significant. However, this moderated mediation was not found in the association of self-esteem with FWB.

## 4. Discussion

To our knowledge, the study is the first to identify the mediating role of SPB and the moderating role of age in accounting for the impact of self-esteem on HRQoL in women with ovarian cancer. Our results show that self-esteem could improve the HRQoL (PWB, EWB, and FWB) partly by reducing SPB in Chinese patients with ovarian cancer. In line with previous findings among cancer survivors across countries [12,13], self-esteem was a significant positive indicator of HRQoL in our sample. This result further supports the notion that self-esteem could help patients effectively manage the adversity caused by cancer and its treatment to mitigate psychological distress [34,35]. People with high self-esteem generally tend to pay attention to their strengths, such as resilience [36] and self-efficacy [37], and actively play the role of these strengths for surviving the challenges of stressful events. In addition, self-esteem can promote patients to express their reasonable needs and create active social communication for obtaining high social support [12,32]. Therefore, the internal psychological resources and external social support and assistance stimulated by high self-esteem could work together to improve the HRQoL of cancer patients.

Also, self-esteem could function as a key psychosocial resource that mitigates SPB through dual pathways. Internally, high self-esteem satisfies core psychological needs for autonomy, competence, and relatedness, buffering the sense of helplessness induced by illness [38]. It fosters positive reappraisal and meaning-focused coping, enabling patients to view challenges as manageable rather than catastrophic, thereby directly reframing the cognitive basis of burden [39,40]. Externally, self-esteem facilitates the social support process. It promotes proactive support-seeking by reducing fears of rejection, and enhances the perceived quality and utilization efficiency of received support by fostering positive interpretations of caregiving [12,39]. This creates a virtuous cycle where effective support receipt alleviates practical stressors, while reciprocal gratitude maintains relationship equity.

This result aligned with previous findings that SPB negatively impacted HRQoL [18,19]. For PWB, SPB acts as a chronic stressor that can heighten the central sensitization to pain and treatment-related symptoms (e.g., fatigue) [41], while its associated stress response may exacerbate inflammatory processes [42], creating a vicious cycle of symptom amplification. Regarding EWB, SPB is a potent direct generator of guilt, shame, and relational anxiety, which consume cognitive resources and inhibit the capacity for positive affect [20]. This leads to a state of emotional depletion characterized by heightened distress and diminished joy or hope. Concerning FWB, SPB triggers maladaptive behavioral patterns, primarily avoidance and withdrawal [43], as patients intentionally curtail activities to minimize their perceived burden on caregivers. This, coupled with the motivational erosion stemming from feeling undeserving of care, results in progressive functional decline and social isolation. Accordingly, the mediation results indicate that ovarian cancer patients with high self-esteem could reduce the perception of burdens to caregivers or families and improve their HRQoL. SPB is common in cancer patients, so how to cope with it is an urgent concern. Our result suggests that self-esteem improvement would be an intervention target for reducing the sense of burden in patients with ovarian cancer.

However, we found a non-significant mediation of SPB between self-esteem and SFWB. SPB may be more directly channeled toward personal and physical health outcomes (like PWB, EWB, and FWB). In contrast, SFWB is profoundly shaped by the quality of family support and external social relationships related to pre-existing family structures and cultural norms [44], which may be less directly contingent on one’s own burden perception, leading to a non-significant mediation. Moreover, threats to SFWB in the context of ovarian cancer (e.g., lack of family support, role strain, and communication difficulties) might be buffered by different factors, such as relationship intimacy, communication patterns, or family adaptability [45], which were not measured in this study.

In addition, this is the first study to demonstrate that age simultaneously moderates the impact of self-esteem on SPB and the impact of SPB on HRQoL in women with ovarian cancer. In younger patients, self-esteem could substantially affect SPB; the latter was more strongly related to PWB and EWB. With the increase in age, the mediation of SPB in the impacts of self-esteem on PWB and EWB gradually disappeared. There could be several central mechanisms to address the moderation of age, involving social roles and functions, psychological resources, reproductive impact, and emotional sensitivity. For younger women, self-esteem and well-being are often closely tied to the acquisition and performance of age-normative social roles, such as partner, mother, and professional. Ovarian cancer disrupts these roles acutely [46], potentially devastating self-esteem and amplifying feelings of being a burden, thereby creating a strong pathway to reduced HRQoL [47]. In contrast, for older women, the salience of these specific roles may diminish, with self-esteem deriving from a broader integration of life experiences and acceptance. Concurrently, the social expectation of receiving care may reduce SPB. Filial piety is an essential Chinese cultural value, and children must support and obey their parents [48]. Children should provide adequate emotional, financial, and caring support when parents become seriously ill. This cultural value may lead to the fact that the HRQoL of older patients is not sensitive to their perceived burdens. Moreover, younger patients can often take good care of themselves, pay attention to their own needs, and live in a self-improvement way [49]. Due to aging and disease, the ability to self-care is significantly reduced, and older people consciously or unconsciously ignore their needs [49]. Younger women with high self-esteem may likely possess and utilize sufficient internal and external resources for coping with cancer, including self-efficacy, resilience and social support, contributing to a lower SPB. In addition, self-esteem can lead to extroverted and acceptance behaviors and is intrinsically linked to social connections. Younger people frequently engage in a variety of interpersonal interactions, and those with high self-esteem can take an active role in these interpersonal connections [50]. While obtaining social support, they could avoid the loneliness caused by interpersonal isolation and frequently feel positive emotions, which is conducive to reducing SPB. With the aging process, older people’s available personal and social resources are gradually reduced [51]. The moderating role of age could also be understood as a confluence of social role transitions and disease-specific, reproductive developmental disruptions. For a young patient, ovarian cancer simultaneously blocks the acquisition of future social roles (e.g., mother) and attacks the biological capacity underpinning those roles [24,25,26]. It double jeopardy magnifies the psychological impact, making self-esteem and SPB potent determinants of HRQoL in this subgroup. In later life, when natural reproductive senescence has occurred, these specific threats attenuate in salience. Age-related differences in emotional processing offer another key mechanism for the moderation [28,29]. Younger adults’ greater sensitivity to threats and lower regulatory capacity can lead them to perceive illness as a catastrophic attack on self-esteem and intensify distress from SPB, thereby strengthening the pathway to reduced HRQoL. In contrast, the enhanced emotion regulation capacities common in later life likely buffer self-esteem and facilitate recovery from SPB, resulting in a weaker observed pathway. Future research is needed to clarify this discrepancy.

As two intervention targets for improving the HRQoL of ovarian cancer patients, family members or caregivers and health care providers can collaboratively motivate patients to develop positive self-evaluation and reduce their cognition of burdensomeness, especially among younger patients. Some interventions for improving self-esteem have been verified in cancer patients’ HRQoL, such as psychoeducational intervention [52] and meaning-making intervention [53]. Women with ovarian cancer should be supported in making personal efforts to adapt to their conditions, including striving to do certain activities alone, looking for positive aspects of life, and avoiding giving in to thoughts of burdensomeness in order to lessen their SPB [54].

A few limitations need to be addressed. First, all patients were recruited from one center of gynecologic oncology therapy. The representativeness of our sample is limited. The findings should only be loosely extrapolated to other ovarian cancer populations with different cultural backgrounds. In cultural contexts like China, where family caregiving norms are strong, the experience of SPB might be particularly pronounced, and the link between self-esteem and SPB could be differently shaped than in individualistic societies [55]. Second, the dynamic changes in self-esteem, SPB, and HRQoL and the causal relationships among the variables cannot be determined because of cross-sectional data. Longitudinal and multicenter studies are warranted to establish causality and improve generalizability. Third, given the impact of female-specific concerns on self-evaluation and perceived burden regarding femininity, sexual attractiveness, and fertility, future studies need to examine how these specific concerns affect HRQoL among ovarian cancer patients. Fourth, the self-reported approach was used to assess self-esteem, SPB, and HRQoL. Potential limitations include recall or reporting biases, which may reduce the reliability and validity of the measurements and underestimate or overestimate their associations. Social desirability bias might lead to the underreporting of psychological distress (like high SPB or low self-esteem), even if some steps were taken to mitigate it (e.g., ensuring anonymity). Moreover, a Harman single-factor test was conducted to assess common method bias. The results, which showed the first unrotated factor explained 28.0% of the variance (below the 40% threshold), did not suggest substantial common method bias. Another potential bias could arise from differences between face-to-face and telephone interviews, although standardization measures (identical instruments, trained interviewers, and pilot testing) were implemented to minimize mode effects that could have influenced responses in ways not captured by our design.

## 5. Conclusions

Self-esteem could improve HRQoL (PWB, EWB, and FWB) partly by reducing SPB in ovarian cancer patients, and the pathway for PWB and EWB is stronger in younger patients. Clinical programs integrating components that strengthen self-esteem and reduce SPB may be particularly beneficial for younger women with ovarian cancer. Longitudinal and multicenter studies are warranted to establish causality and improve generalizability.

## Figures and Tables

**Table 1:** Demographic and clinical characteristics of the subjects.

Variables	*n*	%
Demographic characteristics		
Age (years)		
≤45	35	17.2
46–55	71	35.0
56–65	70	34.5
>65	27	13.3
Marital status		
Married/cohabited	182	89.7
Single/divorced/widowed/separated	27	10.3
Educational level		
Primary school or below	33	16.3
Junior high school	72	35.5
Senior high school	48	23.6
Junior college	25	12.3
College or above	25	12.3
Place of residence		
Urban	130	64.0
Rural	73	36.0
Annual household income (CNY)		
<30,000	72	35.5
<80,000	62	30.5
<120,000	52	25.6
≥120,000	17	8.4
Clinical characteristics		
Disease status		
New onset	179	88.2
Relapse	24	11.8
Duration of suffering from cancer (years)		
≤1	53	26.1
≤2	43	21.2
≤3	49	24.1
≤4	33	16.3
>4	25	12.3
FIGO stage		
Ⅰ	66	32.5
II	43	21.2
III	75	36.9
IV	16	7.9
Not staged	3	1.5
Surgery type		
Minimally invasive surgery	22	10.8
Laparotomy	177	87.2
No operation	4	2.0
Chemotherapy		
Yes	132	65.0
No	71	35.0
Chronic comorbidity		
No	132	65.0
Yes	71	35.0
Family history of cancer		
No	161	79.3
Yes	42	20.7
Pregnancy history		
Yes	190	93.6
No	13	6.4

CNY: Chinese Yuan; FIGO: International Federation of Gynecology and Obstetrics.

**Table 2:** Descriptive statistics and correlations among self-esteem, SPB, and HRQoL.

Variables	Mean (SD)	1	2	3	4	5
1. Self-esteem	33.47 (4.44)	1				
2. SPB	20.63 (10.34)	−0.353 **	1			
3. PWB	23.40 (5.32)	0.451 **	−0.503 **	1		
4. SFWB	19.90 (5.57)	0.216 **	−0.085	0.140 *	1	
5. EWB	20.15 (4.08)	0.364 **	−0.306 **	0.515 **	0.101	1
6. FWB	20.25 (6.24)	0.461 **	−0.387 **	0.524 **	0.577 **	0.367 **

SPB: self-perceived burden; HRQoL: health-related quality of life; PWB: physical well-being; SFWB: social/family well-being; EWB: emotional well-being; FWB: functional well-being; SD: standard deviation. *, *p* < 0.05 (two-tailed), **, *p* < 0.01 (two-tailed).

**Table 3:** Mediating role of SPB in the association between self-esteem and HRQoL.

Dependent Variables	Coefficients				*a* × *b* (95% CI)
	*c*	*a*	*b*	*c′*	
PWB	0.351 **	−0.231 **	−0.322 **	0.276 **	0.074 (0.018, 0.153)
SFWB	0.219 **		−0.011	0.216 **	0.003 (−0.047, 0.041)
EWB	0.348 **		−0.209 **	0.300 **	0.048 (0.001, 0.125)
FWB	0.431 **		−0.244 **	0.375 **	0.056 (0.009, 0.114)

SPB: self-perceived burden; HRQoL: health-related quality of life; PWB: physical well-being; SFWB: social/family well-being; EWB: emotional well-being; FWB: functional well-being; CI; confidence interval. Marital status, place of residence, disease status, duration of suffering from cancer and chronic comorbidity were adjusted. **, *p* < 0.01 (two-tailed).

**Table 4:** Results of moderated mediation.

HRQoL	Age	Mediations	95% CI
PWB			
	Younger	0.197	0.062, 0.397
	Average ^1^	0.076	0.020, 0.154
	Older ^1,2^	0.010	−0.023, 0.065
EWB			
	Younger	0.182	0.043, 0.375
	Average ^1^	0.053	0.006, 0.118
	Older ^1,2^	<0.001	−0.030, 0.027
FWB			
	Younger	0.085	−0.026, 0.236
	Average	0.056	0.010, 0.112
	Older	0.017	−0.049, 0.085

Younger group, −1 SD; Average group, the mean; Older group, +1 SD. Marital status, place of residence, disease status, duration of suffering from cancer and chronic comorbidity were adjusted. HRQoL: health-related quality of life; PWB: physical well-being; EWB: emotional well-being; FWB: functional well-being; CI: confidence interval; SD: standard deviation. ^1^ compared with “Younger group”, *p* < 0.05 (two-tailed); ^2^ compared with “Average group”, *p* < 0.05 (two-tailed).

## Data Availability

The authors confirm that the data supporting the findings are available from the corresponding author upon reasonable request.

## Supplementary Materials

The following supporting information can be downloaded at: https://www.mdpi.com/article/10.3390/curroncol33010023/s1, Table S1: Group differences in SPB and HRQoL.

## Abbreviations

The following abbreviations are used in this manuscript:

HRQoL	Health-related quality of life
SPB	Self-perceived burden
FACT-G	Functional Assessment of Cancer Therapy-General
PWB	Physical well-being
SFWB	Social/family well-being
EWB	Emotional well-being
FWB	Functional well-being
CI	Confidence interval
RSES	Rosenberg Self-Esteem Scale
SPBS	Self-Perceived Burden Scale
CNY	Chinese Yuan
FIGO	International Federation of Gynecology and Obstetrics
SD	Standard deviation
VIF	Variance inflation factor
