# Supplementary material for: Impacts of Self-Esteem and Self-Perceived Burden on Health-Related Quality of Life Among Patients with Ovarian Cancer: Does Age Matter?
Source: Curr Oncol. 2026 Jan 1;33(1):23. doi: 10.3390/curroncol33010023 (PMC12840368; doi:10.3390/curroncol33010023)
Supplement: Supplementary file 1 [file curroncol-33-00023-s001.zip › Table S1.pdf]

Table S1. Group differences in SPB and HRQoL.

Variables	SPB			PWB			SFWB			EWB			FWB		
	Mean (SD)	*F*/*t*	*p*	Mean (SD)	*F*/*t*	*p*	Mean (SD)	*F*/*t*	*p*	Mean (SD)	*F*/*t*	*p*	Mean (SD)	*F*/*t*	*p*
Demographic characteristics															
Age (years)		0.612	0.608		0.308	0.820		0.610	0.609		0.167	0.918		0.442	0.723
≤ 45	20.60 (10.50)			23.56 (6.33)			19.98 (7.11)			19.93 (5.01)			19.57 (7.56)		
46-55	21.77 (11.43)			22.94 (5.65)			19.94 (5.84)			20.12 (4.29)			20.03 (6.04)		
56-65	20.21 (9.56)			23.59 (4.96)			20.32 (4.63)			20.10 (3.73)			20.91 (5.59)		
> 65	18.78 (9.24)			23.94 (3.93)			18.59 (5.70)			20.64 (3.06)			20.00 (6.70)		
Marital status		2.639	0.009		0.370	0.712		2.110	0.036		0.231	0.817		0.378	0.706
Married/cohabited	19.99 (9.89)			23.45 (5.34)			20.18 (5.51)			20.13 (4.08)			20.31 (5.95)		
Single/divorced/widowed/separated	26.19 (12.69)			23.00 (5.27)			17.45 (6.47)			20.35 (4.16)			19.76 (8.52)		
Educational level		2.387	0.052		1.093	0.361		0.856	0.491		0.400	0.808		0.459	0.766
Primary school or below	23.93 (9.56)			21.95 (6.07)			19.93 (5.65)			19.92 (4.22)			19.82 (6.33)		
Junior high school	20.61 (11.56)			23.43 (4.76)			19.48 (5.33)			20.01 (4.55)			20.05 (6.57)		
Senior high school	21.77 (10.27)			23.37 (5.81)			20.42 (5.36)			20.44 (3.12)			21.07 (5.58)		
Junior college	16.40 (7.28)			24.76 (3.92)			21.28 (4.48)			20.86 (2.58)			20.80 (4.62)		
College or above	18.40 (9.06)			23.96 (5.97)			18.69 (7.92)			19.61 (5.30)			19.28 (7.84)		
Place of residence		2.518	0.013		2.432	0.017		2.508	0.013		0.909	0.364		1.991	0.048
Urban	19.28 (10.10)			24.13 (4.67)			20.63 (5.60)			20.35 (4.09)			20.90 (6.30)		
Rural	23.04 (10.40)			22.11 (6.15)			18.58 (5.58)			19.80 (4.05)			19.09 (6.01)		
Annual household income (CNY)		0.582	0.627		1.160	0.326		0.141	0.935		0.077	0.973		0.795	0.498
< 30,000	20.84 (10.86)			22.73 (6.11)			20.24 (5.73)			20.14 (4.21)			20.18 (6.33)		
< 80,000	20.70 (10.37)			23.55 (4.89)			19.69 (6.26)			20.30 (4.08)			19.48 (6.33)		
< 120,000	19.44 (8.69)			23.52 (5.13)			19.66 (4.93)			19.95 (3.39)			21.29 (5.55)		
≥ 120,000	23.18 (12.84)			25.34 (3.31)			19.90 (5.63)			20.29 (5.53)			20.16 (7.57)		
Clinical characteristics															
Disease status		3.701	0.000		2.871	0.008		1.149	0.252		0.979	0.329		0.808	0.420
New onset	19.68 (9.76)			23.94 (4.74)			19.73 (5.66)			20.25 (3.89)			20.38 (6.11)		
Relapse	27.75 (11.92)			19.42 (7.52)			21.14 (5.64)			19.39 (5.28)			19.28 (7.26)		
Duration of suffering from cancer (years)		3.349	0.011		3.033	0.019		0.376	0.826		3.606	0.007		2.827	0.026
≤ 1	24.32 (10.48)			21.35 (5.81)			19.88 (6.70)			18.47 (5.03)			17.94 (7.26)		

≤ 2	21.38 (10.59)			23.57 (5.34)			19.23 (6.06)			20.87 (2.94)			20.42 (6.69)		
≤ 3	18.14 (9.58)			24.31 (4.88)			20.42 (5.09)			21.20 (3.04)			21.63 (5.48)		
≤ 4	20.11 (9.99)			24.75 (5.00)			20.43 (4.82)			20.11 (4.13)			21.42 (5.31)		
> 4	17.11 (9.59)			23.92 (4.55)			19.36 (4.89)			20.48 (4.37)			20.60 (4.52)		
FIGO stage		2.625	0.052		4.017	0.008		0.295	0.829		1.809	0.147		0.626	0.599
Ⅰ	17.96 (9.10)			25.22 (3.43)			20.27 (5.95)			20.98 (3.46)			20.98 (5.88)		
Ⅱ	22.18 (10.48)			22.37 (6.16)			19.81 (5.07)			19.94 (3.98)			19.91 (6.55)		
Ⅲ	21.08 (11.15)			22.61 (5.74)			19.37 (5.71)			19.40 (4.79)			19.61 (6.25)		
Ⅳ	24.44 (8.90)			22.31 (6.21)			19.68 (5.76)			20.38 (2.60)			19.69 (6.69)		
Surgery type		0.261	0.794		0.676	0.500		0.318	0.751		0.309	0.758		0.123	0.902
Minimally invasive surgery	21.00 (11.35)			24.24 (4.19)			19.56 (5.07)			19.91 (4.22)			20.41 (6.22)		
Open surgery	20.40 (10.03)			23.46 (5.25)			19.96 (5.67)			20.20 (4.09)			20.24 (6.21)		
Chemotherapy		1.908	0.058		1.788	0.075		0.233	0.816		1.096	0.274		0.047	0.963
Yes	21.64 (10.82)			22.96 (5.83)			19.97 (5.34)			19.92 (4.18)			20.24 (6.47)		
No	18.76 (9.17)			24.22 (4.13)			19.77 (6.26)			20.58 (3.87)			20.28 (5.84)		
Chronic comorbidity		2.145	0.033		2.506	0.014		0.965	0.336		0.922	0.358		2.214	0.028
No	19.50 (9.91)			24.12 (4.90)			19.62 (5.83)			20.34 (4.20)			20.96 (6.00)		
Yes	22.74 (10.87)			22.08 (5.85)			20.42 (5.34)			19.79 (3.84)			18.94 (6.52)		
Family history of cancer		1.178	0.240		2.106	0.036		0.811	0.418		0.406	0.685		0.124	0.901
No	20.20 (10.40)			23.80 (5.20)			19.73 (5.41)			20.09 (4.26)			20.22 (6.10)		
Yes	22.31 (10.08)			21.88 (5.58)			20.53 (6.59)			20.38 (3.31)			20.36 (6.85)		
Pregnancy history		1.116	0.266		0.471	0.638		1.255	0.232		0.207	0.836		1.393	0.165
Yes	20.85 (10.49)			23.36 (5.41)			20.10 (5.34)			20.17 (4.10)			20.41 (6.08)		
No	17.54 (7.50)			24.08 (3.93)			16.92 (9.02)			19.92 (3.86)			17.92 (8.21)		

SPB: self-perceived burden; HRQoL: health-related quality of life; SD: standard deviation; PWB: physical well-being; SFWB: social/family well-being; EWB: emotional well-being; FWB: functional well-being; CNY: Chinese Yuan; FIGO: International Federation of Gynecology and Obstetrics.
